# Slobbering

**Published:** 1896-06

**Authors:** 


### Slobbering.

Dr. Sauchez de Silvera, we learn from *La Sperimentale,* has not thought this homely subject unworthy of study, and he has written an essay upon it. From the observations of Dr. Couetoux, of Nantes, and his own, he concludes that healthy infants never dribble; that infants which dribble only in the day time, though apparently in good health, have their digestive functions impaired; that infants which dribble at night are suffering from obstruction of nasal respiration to a greater or less degree; that these phenomena are altogether unconnected with dentition.

